# Experiences of workplace breastfeeding in a provincial government setting: a qualitative exploratory study among managers and mothers in South Africa

**DOI:** 10.1186/s13006-020-00342-4

**Published:** 2020-11-23

**Authors:** Bongekile P. Mabaso, Ameeta Jaga, Tanya Doherty

**Affiliations:** 1 Department of School of Management Studies, University of Cape Town, Cape Town, South Africa; 2 Health Systems Research Unit, South African Medical Research Council, Cape Town, South Africa; 3 Department of Paediatrics and Child Health, Faculty of Health Sciences, University of Cape Town, Cape Town, South Africa

**Keywords:** Breastfeeding at work, Workplace support, South Africa, Qualitative exploratory, Work-family, Provincial government

## Abstract

**Background:**

Return to employment is a major barrier to breastfeeding continuation, globally and in the Southern African context. The Lancet Breastfeeding Series revealed an explicit need for research exploring breastfeeding as a workplace issue in low- and middle-income countries. A dearth of research on workplace breastfeeding in South Africa calls for attention to this topic. This study sought to explore breastfeeding at work experiences from the perspective of employed mothers and senior managers in a provincial government setting in South Africa.

**Methods:**

The study adopted an exploratory qualitative design with multi-perspective semi-structured interviews. Snowball sampling was employed to recruit twelve participants, senior managers (*n* = 4) and employed mothers (*n* = 8), from two provincial government departments in Cape Town, South Africa. Interviews were conducted between April and August 2018 to capture participants’ experiences with breastfeeding in the workplace. Thematic analysis was used to analyse data.

**Results:**

Four key themes that described experiences of workplace breastfeeding emerged which further traversed three critical maternity periods: pregnancy, maternity leave, and return to work. The prevalent themes were: 1) Knowledge about the legislation and breastfeeding support benefits. Most participants only knew about the legislated four months maternity leave and time off for prenatal visits but lacked knowledge about comprehensive maternity benefits; 2) Perceptions and experiences of breastfeeding in the workplace. Breastfeeding was perceived to be a mother’s responsibility and a private issue. As a result, most participants stopped breastfeeding prior to or immediately upon return to work after maternity leave; 3) Barriers to breastfeeding continuation, such as the absence of a conversation about infant feeding plans between managers and mothers; and 4) Recommendations to improve breastfeeding support at work from an individual, organisational and national level.

**Conclusions:**

Our study contributions emphasise that breastfeeding support from managers should begin prior to the mother taking maternity leave, and that in addition to providing supportive facilities (such as private space and breastmilk storage), immediate supervisor support may be critical in fostering breastfeeding-friendly workplaces for mothers. Management implications for advancing workplace breastfeeding support in the public sector are presented.

## Background

Breastfeeding is a key child survival strategy to prevent childhood illnesses and deaths. Positive economic and environmental changes and improved maternal health have also been associated with breastfeeding [1]. Current data on breastfeeding practices in South Africa shows that 93% of mothers initiate breastfeeding within the first hour of birth, but that only 24% of infants are breastfed exclusively by age four-five months [2, 3]. South Africa has had a history of the lowest exclusive breastfeeding rates in the world at 8 % between 1998 and 2012 [2]. While improvements have been made [4], the progress is still too slow and far from the Global Nutrition target of 50% by 2025 [5].

Return to employment is a major reason breastfeeding is compromised both globally [6–9] and in the Southern African context [10, 11]. Not surprising, breastfeeding at work is a complex work-family issue because in order to maintain the World Health Organisation’s (WHO) recommendation to breastfeed exclusively for the first six months of the infant’s life [12], most mothers must engage in this responsibility in the time and space of paid work. Employed mothers tend to stop breastfeeding in preparation to return to workplaces that are not conducive to maternal needs [13, 14] and breastfeeding mothers often fall short of the ideal worker ideology around which organisations are built. Ideal workers single-mindedly devote their efforts to organisational goals [15], not allowing distractions such as reproductive needs which are experienced by pregnant and breastfeeding mothers [14]. Workplace breastfeeding support could make positive organisational contributions by decreasing absenteeism, job dissatisfaction, staff turnover and improve staff retention [16–18].

In South Africa, since the first democratic elections in 1994, there has been an increase in the number of females in the labour force because of supportive legislative policies that promoted access to education and employment for women. According to the 2019 Quarterly Labour Force Survey (April – June 2019) 44.6% of the employed labour force were female, of which most (30.7%) were in the formal sector [19]. This increased female representation [20] has focused the government’s attention on laws that promote gender equality, such as 17 weeks unpaid maternity leave with a subsidy of 66% of the mother’s salary that can be claimed from the Unemployment Insurance Fund [21]. With the optimal infant feeding recommendation being six months exclusive breastfeeding [12], mothers returning to work at four months or earlier would need to breastfeed or express breast milk whilst in the workplace. Thus, the state also provides for legislated breastfeeding breaks at work, for 30 min twice per working day for the first six months of the child’s life [22]. Unfortunately, the limited available literature has shown great ignorance and poor enforcement of the legislated maternity protection by South African employers, particularly regarding breastfeeding breaks [23].

Current workplace breastfeeding literature is mostly from high income countries [24–27]. Research from low- and middle-income countries in the Global South are lacking in providing a contextually rich understanding of employed mothers’ breastfeeding practices and salient forms of support to advance breastfeeding at work. Such nuanced insights are needed to inform context relevant interventions [28–30]. The aim of this study is to explore experiences of workplace breastfeeding among employees and managers in a provincial government setting in South Africa, to gain an understanding of the local specificities that shape this phenomenon. This in turn could inform appropriate interventions for creating breastfeeding supportive workplaces that meet these mothers’ needs and advance the international body of literature.

## Methods

### Study design

The study adopted an exploratory qualitative design. Multi-perspective semi-structured interviews were conducted face-to-face with senior managers and mothers who worked in a provincial government setting in South Africa. The use of a multi-perspective approach was effective for triangulation and ensuring a richer understanding of the subject as senior managers create the workplace culture which influences the availability and uptake of workplace practices and mothers’ behaviours [31, 32].

### Study setting and participants

The study was conducted in Cape Town, a metropolitan city in the Western Cape province with a population of 4,232,276 (64% of the total provincial population). The Western Cape province is the third largest (by population) of nine provinces in South Africa. Cape Town was selected as the study site for its status as the legislative capital in which government head offices, key policymakers, and several public departments are located [33]. The provincial government setting was purposely selected because it serves as a model employer in that it provides fully paid maternity leave for the legislated four months. In addition, provincial government is the largest employer of all government sectors nationally and has a workforce of 81,000 in the Western Cape, of which 52% are women. Two of the 13 departments in this provincial government participated in the study. The inclusion criterion for managers was that they had supervised pregnant employees who returned to the same workplace after maternity leave. For mothers, those who had babies and returned to the same workplace and were breastfeeding or had breastfed their infants in the past two years were eligible to participate in the study. Emails were sent to heads of departments inviting eligible employees to participate. With no replies to the invitation, the first few participants were then found through contacts of provincial government employees who were part of a larger research project in this area. Thereafter we relied on snowball sampling and concluded sampling upon reaching data saturation [34].

### Data collection

Semi-structured interviews were conducted between April and August 2018 by a small team of interviewers, BPM or AJ, a scribe and an observer. In a conversational manner, participants were asked to share personal breastfeeding experiences or experiences of supporting breastfeeding employee(s) upon return to work post maternity leave, as well as how they thought that support for breastfeeding at work could be improved. For example, mothers were asked “can you tell me about how you fed your child when you returned to work after maternity leave?” while managers were asked “tell me about your experiences of being a manager to women returning from maternity leave?”. All interviews were conducted in English and interview arrangements were made with participants directly. Each interview lasted between 30 to 77 min. Scheduling interviews was challenging given the nature of the participants’ work roles, especially those in senior management, and their last-minute cancellations often required rescheduling of interviews. The nature of their work roles cannot be detailed to protect the identification of the departments that participated in the study. All interviews were conducted at the participants’ workplaces, as indicated by each to be the most convenient. Basic demographic data, that is, race, level of education, and age were collected to describe the sample.

To enhance trustworthiness, after each interview a de-briefing meeting was held with the interviewing team to discuss emerging ideas and to assess if follow up questions with participants where necessary. As a result, and as part of member checking, three senior managers and mothers were interviewed a second time to gain further clarity and to ensure that the study findings reflected their views [34].

### Data analysis

**Fig. 1:**
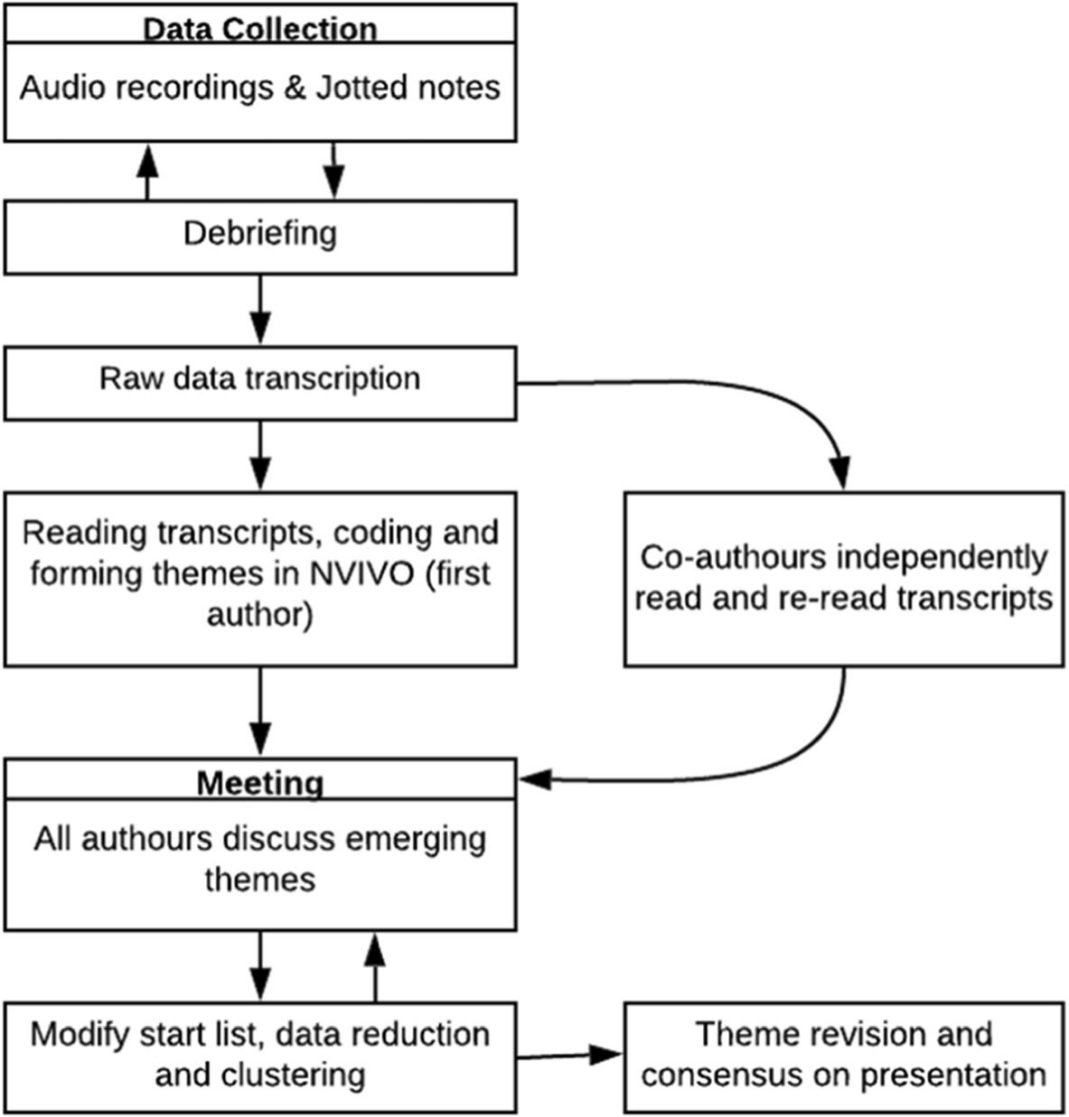
The data collection and analysis process

Preliminary data analysis occurred concurrently with data collection (Fig. 1). All audio recordings were transcribed verbatim by a professional transcription company and pseudonyms were used for participants’ names and departments. Raw data were imported into NVIVO 12 Pro, a computer software program used to manage qualitative data [35]. Inductive thematic analysis was used where through multiple readings of the transcripts the first author generated codes and then themes which emerged from the participants’ responses, rather than from a priori themes from theory [36]. The second and third author independently read and coded the interview transcripts to establish patterns in the data and together all the authors critically discussed their findings, merging or renaming subthemes, and refining the themes until they reached consensus [37]. A final set of four themes that enhanced our understanding of workplace breastfeeding were agreed upon: 1) Knowledge about the legislation and breastfeeding support benefits; 2) Perceptions and experiences of breastfeeding in the workplace; 3) Barriers to breastfeeding continuation; and 4) Recommendations to improve workplace breastfeeding support. Moreover, consensus was reached by all the authors to organise the findings into distinct, yet inter-related themes and subthemes, in a novel way across three critical time phases: pregnancy, maternity leave, and the return to work (Table 1), emphasising that breastfeeding support from managers should begin prior to the mother taking maternity leave.

**Table 1:** Emerging themes and sub-themes on breastfeeding at work experiences across maternity phases in the provincial government sector

	Theme 1: Knowledge about the legislation and breastfeeding support benefits	Theme 2: Perceptions and experiences of breastfeeding in the workplace	Theme 3: Barriers to breastfeeding continuation	Theme 4: Recommendations to improve breastfeeding support at work
**Pregnancy** [sub-themes]	• Knowledge about maternity leave uptake. • Poor knowledge about breastfeeding breaks. • Optimal knowledge about benefits of supporting breastfeeding.	• Breastfeeding considered a private and personal matter. • Support dependant on managers’ personal values and experiences.	• Absence of conversation about breastfeeding. • Confusion on who is responsible for initiating breastfeeding support conversation at work. • Manager’s gender consideration on breastfeeding support. • Lack of instrumental support at work.	• Raise awareness about the maternity support legislation. • Have a workplace breastfeeding policy. • Provide instrumental support. • Breastfeeding specific social support to be provided.
**Maternity** [sub-themes]	• Optimal knowledge about benefits of supporting breastfeeding.	• Breastfeeding considered a private and personal matter. • Early breastfeeding cessation.	• Stress associated with transitioning back to employment.	• Raise awareness about the maternity support legislation. • Extension of maternity leave. • Breastfeeding specific social support to be provided.
**Return to work** [sub-themes]	• Poor knowledge about breastfeeding breaks. • Optimal knowledge about benefits of supporting breastfeeding.	• Difficulty expressing at work. • Early breastfeeding cessation. • Breastfeeding considered a private and personal matter. • Support dependant on managers’ personal values and experiences.	• Absence of conversation about breastfeeding. • Confusion on who is responsible for initiating breastfeeding support conversation at work. • Manager’s gender consideration on breastfeeding support. • Influence of job characteristics. • Stress associated with transitioning back to employment. • Lack of instrumental support at work.	• Have a workplace breastfeeding policy. • Provide instrumental support. • Extension of maternity leave. • Breastfeeding specific social support to be provided.

### Ethics

Ethics approval was obtained from the University of Cape Town Commerce Faculty Ethics in Research Committee in April 2018 [REC 2018/004/013] and from the two government departments. Participation in the study was voluntary and responses were kept anonymous and confidential. Written informed consent was obtained and interviews were audio recorded with permission from all the respondents.

## Results

Participants were diverse in race, level of education, and age. All except one participant had post graduate qualifications and held professional positions. Three of the four managers were male, and all the managers had children (Table 2).

**Table 2:** Demographic characteristics of study participants

Participant	Level of Education	Gender	Race	Age	Work Position
Mother 1	Postgraduate	F	White	38	Senior manager
Mother 2	Postgraduate	F	Coloured^a^	32	Project Manager
Mother 3	High School	F	Coloured	40	Admin Clerk
Mother 4	Postgraduate	F	Coloured	36	Supply Chain Officer
Mother 5	Postgraduate	F	African	36	Educator
Mother 6	Postgraduate	F	Coloured	34	Educator
Mother 7	Postgraduate	F	Coloured	31	Educator
Mother 8	Postgraduate	F	Coloured	31	Monitoring Officer
Manager 1	Postgraduate	M	Coloured	47	Senior manager
Manager 2	Postgraduate	M	African	55	Chief Director
Manager 3	Postgraduate	F	White	59	Director
Manager 4	Postgraduate	M	Coloured	49	Director

^a^In Southern Africa, the term ‘coloured’ denotes a person of mixed racial ancestry and is used officially in South Africa’s national statistics [38]

### Pregnancy phase

#### Pregnancy perceived as a mother’s issue

Senior managers and mothers generally perceived pregnancy as private and a mother’s issue, that needed to be concealed from work processes. None of the mothers had any infant feeding related conversation with their managers prior to going on maternity leave. Most mothers did not even consider combining breastfeeding and work, while managers distanced themselves and felt that only if mothers made enough of a demand, would there be a need to address pregnancy and breastfeeding issues at work. Some participant’s views are expressed in the following quotes:

“Actually no [conversation about breastfeeding], the only discussion we have had in terms of that, is okay, what plans and arrangements are you going to make when the maternity leave is [over] . . . who is going to look after the baby, and those kind of things. I don’t know if every woman is comfortable discussing their personal matters like that especially with a male manager . . . but I have never had a discussion, maybe it is something I need to think about going forward.” (Manager 1)

“As long as I told them that I am planning on taking my maternity leave, so I think that was enough for me.” (Mother 3)

“I think the conversation ended there after me telling them I am expecting a baby. There was no conversations about how I am going to handle the pregnancy or whatever or breastfeeding later on, so no conversations like that”. (Mother 5)

#### Minimal discussion about maternity benefits with pregnant employees

Most mothers did not have knowledge of their full maternity protection rights such as maternity leave extension or breastfeeding breaks (Basic Conditions of Employment Act, Code of Good Practice), but only knew about the legislated four months maternity leave and time off for prenatal visits. Managers were also not aware that breastfeeding breaks were legislated and assumed that mothers were informed of their maternity benefits. One manager said:

“. . . even the Basic Condition of Employment Act does not have that [breastfeeding breaks]. If it does, I will be surprised because the Basic Condition lays the basic minimum . . . but, definitely there is no break there, I think it is the tea break . . . and smoke breaks is there but not breastfeeding.” (Manager 2)

Only a few mothers reported knowledge of the option to apply for a maternity leave extension to six months. Some who were aware of it, shared that they did not apply for it in fear of their manager thinking that they were attempting to take advantage of the circumstances, as maternity leave tended to be viewed as a holiday. As one mother mentioned:

“I didn’t [have any conversation about the possibility of extending maternity leave]. I must admit I was actually dying to have that conversation with my boss, but I also didn’t want to give that impression that I am trying to ride this leave out, or take advantage of the situation.” (Mother 5)

Some mothers chose not to apply for maternity leave extension because it was unpaid and there was a financial necessity to return to work. While others described doing their own research, most commonly on the internet, to find out information about their maternity rights, such as breastfeeding breaks:

“I just Googled breastfeeding . . . I think it was the Basic Conditions of Employment Act where they say you get two sessions [breastfeeding breaks], thirty minutes each . . . I went and found the policy and so I took it upon myself to educate them [managers] around what the policies are because I did feel conscious that I am going to express and it is going to take time.” (Mother 4)

### Maternity leave phase

#### Preparation for early breastfeeding cessation

Most mothers shared that during their maternity leave they started to become anxious thinking about how to provide optimal nutrition for their infants after returning to work. This anxiety stemmed from several sources including: (1) the lack of decent space to express breast milk at work and adequate storage for mother’s milk, (2) working with painful breasts, and (3) commuting for hours with their expressed milk. Consequently, most mothers decided to wean their infants before or shortly after returning to employment as shared by one mother:

“I just sorted it out myself before I came back to work because I knew I was going to work . . . I didn’t want to feel uncomfortable and now you are working and suddenly there is a wet patch. I decided to do that [stop breastfeeding and started the baby on infant formula] before I went to work, so by the time I returned to work there was nothing [no milk leaking]”. (Mother 7)

A few mothers made plans during their maternity leave to maintain exclusive breastfeeding and started storing milk for when they were back at work:

“So, I started probably two or so weeks before I came back and I started expressing and freezing milk. So, we had a backup supply in the freezer and then obviously I ensured that there was at least four bottles for him in the fridge and then during the day at work I expressed two bottles.” (Mother 8)

### Return to work phase

#### Stress from juggling work and breastfeeding needs

Upon return to employment mothers reported stress dealing with conflict between work demands and their infant’s breastfeeding needs. Here mothers spoke about expectations to be efficient at work whilst experiencing discomfort from full breasts because of a lack of facilities to express mother’s milk:

“I remember also going home every day with really sore breasts because I wasn’t expressing at work and so the milk was building up and building up and it becomes so full and so painful and I also thought this time around [with a new baby] I wouldn’t want to go through that again because it is embarrassing if it breaks through and milk spilling out . . . you come to work and [put a] smile on your face and go on working as normal but you are actually there with full breasts and in pain*.*” (Mother 5)

Another mother added that there is no recognition that a mother returning to work may be breastfeeding and having both work and breastfeeding duties during her work day. She suggested that support would be welcomed to alleviate this pressure that she faced when returning to work:

“I will say that the work place must make that provision for a mother when she comes back to have that place or a room where they can [express]. You can’t breastfeed because the baby is not here but just to make that provision that you can do all your things because I don’t think there is any places that you can as a mother go and sit and even if you come back, no one is talking to you to ask if you are breastfeeding or just to assist you when you come back . . . you just come back in the same situation that you left and so no one is actually talking to you to ask you or even suggest. I will say a person [supervisor] must come to you and say you have a baby now and it is four months, is there certain things that you want or ask you if you are breastfeeding or even ask you if you want to leave earlier but there is no things like that”. (Mother 4)

Juxtaposing this perspective, managers were not aware of this conflict and the breastfeeding related needs of mothers while at work. They perceived that because mothers had not raised the topic of breastfeeding at work, nor requested support in any form, that it was unlikely to be a real concern. As expressed by one senior manager:

“If women are not putting a request or a demand for breastfeeding facilities it will never see the light of the day because other things that are on the table competing for available resources will get priority . . . I cannot remember any union having brought this as a request. I cannot remember. I have been here now for 16 years. I cannot remember any supervisors bringing this as an issue that needs to be looked at.” (Manager 2)

#### Workplace supportive breastfeeding facilities

None of the work spaces in the two departments provided supportive facilities such as a private space, or a fridge for storing milk, for mothers to breastfeed or express milk during the workday. One manager shared:

“This is a very unfortunate part because the government doesn’t have [facilities] and in most offices there is no infrastructure for breastfeeding . . . I think that is the disadvantage for young mothers because we do wonder most of them are highly qualified and they are professional people and that is putting a huge disadvantage, so they must find alternative means [to provide breastmilk] because they just can’t bring babies to work even if they wanted to there are just no facilities.” (Manager 2)

Another manager, while speaking about supportive facilities, was also indirectly suggesting that its solely the mother’s responsibility to find a solution to combine breastfeeding and work:

"I think it’s a bit difficult at work remember because we need to keep it [breast milk] here in the freezer and that is not always available and so on. So, when you are back at work it’s more difficult so you [the mother] will have to find the balance". (Manager 3)

One government department (not part of this study), had a breastfeeding room that was offered to mothers from other departments to use. However, it was often not a logistically viable option to access during the 30 min breastfeeding break as it was in a different building. While few mothers could use this facility enabling their continued breastfeeding, other mothers used the bathroom or offices to express:

“I think what motivated me to stop [breastfeeding] besides the fact that I didn’t have much milk was the fact that the toilet I was using, they were renovating them and so I did not have anywhere else to express.” (Mother 3)

#### Job characteristics and social supportive enablers

Few mothers reported to have continued breastfeeding exclusively after returning to employment and stated that supportive supervisors and co-workers were important enablers:

“The rest of the people [colleagues] were absolutely brilliant and if they knew I was pumping then they would make sure no one would come in and that kind of thing, so they respected that and everything and they were just supportive.” (Mother 1)

“My job requires me to be out in the field and travel a lot and he [supervisor] actually said he understands that and he is limiting my operations to day trips so in the metro and the winelands so I don’t have to sleep over in the west coast and stuff like that. So in terms of his overall [support] he is very accommodating.” (Mother 8)

On the other hand, some mothers shared that co-workers were resentful of mothers who expressed at work, suggesting that they were ‘avoiding work’. Mothers in more senior positions who maintained exclusive breastfeeding reported that their seniority and associated levels of autonomy might have influenced their agency to demand support from their managers.

None of the mothers knew anyone who had breastfed at their workplaces and thus had no positive role models. However, a few managers expressed willingness to offer flexible arrangements to accommodate mothers returning from maternity leave, as captured in the following quotes:

“I am open to the idea of it [providing support to breastfeeding employees] and I don’t have an issue if you need to express”. (Manager 1)

“. . . from where I sit, I have now become aware so it is actually a management responsibility . . . and then sensitization of managers in particular because I think once managers are sensitized on this then it becomes easier because I for example would prefer working through my managers to say but you must support . . . it requires an attitude change how this is actually being implemented . . . I think that can be done through training particularly targeting managers and making sure that they understand the particular needs so is it for example something that can be made available temporarily, what do you need in order for that to happen and so on.” (Manager 4)

“I think if the employers can be more, I don’t know if the word is sensitive but more understanding or accommodating. Yes, if they can be more accommodating in that regard, that would be helpful so providing facilities and obviously be accommodating in terms of allowing you to go and do your expressing at that time . . . I think if they could be more sensitive or maybe do some research of their own”. (Mother 5)

## Discussion

This study provides important new insights into the experiences of workplace breastfeeding among mothers and managers, in a provincial government context in South Africa, shedding light on the needed advancement in workplace breastfeeding support in this middle income context. The study revealed that workplace breastfeeding support should be initiated from pregnancy through maternity leave until after returning to employment. Most workplace breastfeeding literature has focussed on support when the mother has returned to employment, but our findings suggest that this is too late. In the absence of any conversation with managers on reconciling work and breastfeeding prior to the mother going on maternity leave, many mothers tend to cease breastfeeding during maternity leave in preparation to return to work [7, 10, 39]. Our findings suggest that if managers and mothers have a conversation about entitled breastfeeding breaks and workplace support prior to the mother’s maternity leave, it is likely that fewer mothers will perceive return to work as a reason to stop breastfeeding. These conversations if seen as part of a manager’s role in planning the maternity leave arrangements with the mother, may also shift the perspective that breastfeeding is in fact a workplace concern, rather than a mother’s private issue.

Researchers have argued that the perception of breastfeeding as a personal issue is consistent with the view of breasts as private sexual objects. Breastfeeding is associated with an emotional, leaky body that is in conflict with the ideal worker ideology [40–42]. The disapproval of breasts in public spaces leads to feelings of discomfort and shame which when combined with non-supportive workplaces result in early breastfeeding cessation as was evident in this study [43]. Consistent with previous research, managers in this study were not aware that some mothers had a need to breastfeed or express milk during their workday, attesting to the hegemonic masculinity of workplace norms. Many managers left the responsibility to request breastfeeding support to the already overburdened and vulnerable mothers [25], but most mothers were too afraid to ‘rock the boat’ and ask for support, even when they were entitled to breastfeeding breaks. These mothers were therefore unable to take advantage of their full maternity benefits [27]. A study in the United State found that it was unlikely that mothers would request support at non-supportive workplaces creating a false belief that there is no demand nor need for breastfeeding support. The passive tendency to expect mothers to demand support creates a vicious cycle where mothers hold back out of fear of negative consequence(s) and keep breastfeeding needs a secret, while managers withhold support maintaining an unconducive work environment for mothers’ breastfeeding needs [44]. Our study confirms these findings that mothers lacked confidence to request support, despite most mothers in this study being highly educated and holding professional jobs.

Similar to findings from a South African survey [23], both managers and mothers lacked knowledge of the legislation on breastfeeding breaks and maternity protection offered by the employer. Only a few self-informed mothers had researched the full benefits available to them as government employees [39, 45]. Poor knowledge dissemination within the provincial government context and a bureaucratic system that impedes creativity in policy adoption, could contribute to poor implementation of breastfeeding legislation at work [23]. Interestingly, most managers in the study expressed a sincere willingness to support mothers to breastfeed at work, but also clarified that they would require sound training to provide appropriate support, especially since most managers were men. Managers indicated that they were unsure as to what support to provide and how. Furthermore, the study findings suggest that immediate supervisors of pregnant and breastfeeding women (more so than senior management) should be champions for change. Existing research shows that immediate supervisors have greater autonomy to control the day-to-day utilisation of resources, to implement policies and restructure the work time and place for meeting employee needs, compared to senior managers who are more distant from employees’ daily activities [25–27]. As a result, formal breastfeeding at work programs without supervisor support have been shown to result in poor uptake of work-family benefits [46]. Similarly, research shows that mothers thought empathetic supervisors would ease their stress and enable access to maternity benefits, reducing the pressure placed on them to initiate conversations about workplace breastfeeding [26].

Anticipated work-family conflict [47, 48] experienced by mothers during maternity leave about combining breastfeeding and work demands contributed to them weaning their infants sooner than six months. Socio-structural barriers such as work travel and lack of supportive facilities at work contributed to increased anxieties and feelings of not being supported to maintain breastfeeding. We suggest that employers and Human Resource departments provide supportive information during maternity leave to ease the stress experienced by mothers during this phase. Similar to existing studies, we recommend increasing awareness about the legislation and enforcing its implementation in line with good employment practice [25, 49]. For improved workplace support, suggestions for management include: First, developing a written breastfeeding at work policy in consultation with the mothers to ensure that their needs are met by the policy. This policy should be well communicated through various channels to all employees (not just mothers). A breastfeeding policy establishes a positive attitude towards breastfeeding at work and indicates commitment to supporting, protecting and promoting breastfeeding by the organisation aiding breastfeeding continuation [50]. Second, identifying private spaces with appropriate breast milk storage to create a conducive space for mothers who need to express breastmilk. Third, training supervisors to raise their awareness about mothers’ needs to breastfeeding at work, maternity protection legislation, how to foster a positive attitude toward workplace breastfeeding support and equip them with the skills to offer appropriate support. Last, providing flexible work arrangements and on-site childcare to mothers who are navigating breastfeeding and employment [51]. Since the government already has paid maternity leave for four months, it could consider extending paid maternity leave to six months to moderate income loss stress and encourage exclusive breastfeeding.

### Limitations

The use of workspaces for interviews might have restricted the mothers’ openness about negative experiences they might have had in their workplaces. However, the researchers were trained in qualitative research and probed often to get more information from participants and read their body language for signs of discomfort. Experiences from mothers in lower job positions might have been missed as the majority of study participants held professional jobs. Transferability and application of the findings should be limited to countries with similar demographics and national maternity benefits.

## Conclusions

This study revealed that breastfeeding needs to be viewed as an important workplace issue. The main findings from this study were that comprehensive support initiatives should begin as early as pregnancy and continue once the mother returns to employment. Also, conversations between pregnant mothers and their immediate supervisors about how the workplace supports breastfeeding mothers after returning to work would be beneficial in fostering breastfeeding friendly workplaces. Further in-depth research is needed to explore the explicit nature of supervisor support and the supervisor’s needs for appropriately supporting pregnant and breastfeeding mothers regarding breastfeeding at work. This study has provided new insights on workplace breastfeeding support in a provincial government context from a middle-income country marked by low rates of breastfeeding. The findings can inform strategies for employers and policy makers towards creating supportive workplaces for breastfeeding.

## Data Availability

The datasets used and analysed during the current study are available from the corresponding author upon reasonable request.

## Abbreviations

WHO: World Health Organisation
WCG: Western Cape Government

## References

[1] Rollins NC, Bhandari N, Hajeebhoy N, Horton S, Lutter CK, Martines JC (2016). Why invest, and what it will take to improve breastfeeding practices?. Lancet.

[2] Shisana O, Labadarios D, Rehle T, Simbayi L, Zuma K, Dhansay A (2013). South African National Health and nutrition examination survey (SANHANES-1).

[3] South African Demographic & Health Survey. *Key Indicator Report 2016* Available from: http://www.statssa.gov.za/publications/Report%2003-00-09/Report%2003-00-092016.pdf. Accessed 08 Oct 2020.

[4] Jackson D, Swanevelder S, Doherty T, Lombard C, Bhardwaj S, Goga A (2019). Changes in rates of early exclusive breast feeding in South Africa from 2010 to 2013: data from three national surveys before and during implementation of a change in national breastfeeding policy. BMJ Open.

[5] World Health Organization. Nutrition: Global Targets 2025. 2018. Available from: http://www.who.int/nutrition/global-target-2025/en/. Accessed 08 Oct 2020.

[6] Cooklin AR, Rowe HJ, Fisher JRW (2012). Paid parental leave supports breastfeeding and mother-infant relationship: a prospective investigation of maternal postpartum employment. Aust N Z J Public Health.

[7] Chatterji P, Frick KD (2005). Does returning to work after childbirth affect breastfeeding practices?. Rev Econ Househ.

[8] Weber D, Janson A, Nolan M, Wen LM, Rissel C (2011). Female employees' perceptions of organisational support for breastfeeding at work: findings from an Australian health service workplace. Int Breastfeed J.

[9] Kavle JA, LaCroix E, Dau H, Engmann C (2017). Addressing barriers to exclusive breast-feeding in low-and middle-income countries: a systematic review and programmatic implications. Public Health Nutr.

[10] Wainaina C, Wanjohi M, Wekesah F, Woolhead G, Kimani-Murage E (2018). Exploring the experiences of middle income mothers in practicing exclusive breastfeeding in Nairobi, Kenya. Mat Child Health J.

[11] Siziba LP, Jerling J, Hanekom SM, Wentzel-Viljoen E (2015). Low rates of exclusive breastfeeding are still evident in four south African provinces. South African J Clin Nutri.

[12] World Health Organization. *The World Health Organization's infant feeding recommendation 2018*. Available from: http://www.who.int/nutrition/topics/infantfeeding_recommendation/en/. Accessed 08 Oct 2020.

[13] Cahusac E, Kanji S (2014). Giving up: how gendered organizational cultures push mothers out. Gender, Work & Organization..

[14] Gatrell CJ (2007). Secrets and lies: breastfeeding and professional paid work. Soc Sci Med.

[15] Acker J (1990). Hierarchies, jobs, bodies: a theory of gendered organizations. Gend Soc.

[16] Cohen R, Mrtek MB, Mrtek RG (1995). Comparison of maternal absenteeism and infant illness rates among breast-feeding and formula-feeding women in two corporations. Am J Health Promot.

[17] Horta BL, Loret de Mola C, Victora CG (2015). Breastfeeding and intelligence: a systematic review and meta-analysis. Acta Paediatr.

[18] Waite WM, Christakis D (2015). Relationship of maternal perceptions of workplace breastfeeding support and job satisfaction. Breastfeed Med.

[19] Statistics South Africa. *Quarterly Labour Force Survey, Quarter 2: 2019*. 2019 Available from: http://www.statssa.gov.za/publications/P0211/P02112ndQuarter2019.pdf. Accessed 08 Oct 2020.

[20] Casale D, Posel D (2002). The continued feminisation of the labour force in South Africa: an analysis of recent data and trends 1. South African J Econ.

[21] South African Department of Labour. *Unemployment Insurance Fund (UIF) 2018* Available from: http://www.labour.gov.za/DOL/legislation/acts/how-tos/unemployment-insurance-fund-uif/. Accessed 08 Oct 2020.

[22] Government Gazette. Basic Conditions of Employment Amendment Act 7 of 2018. Republic of South Africa. Cape Town: Government Printers; 2018. p. 1–17. Available from: https://www.gov.za/sites/default/files/gcis_document/201811/42059gon1302act7of2018.pdf. Accessed 08 Oct 2020.

[23] Martin-Wiesner P (2018). A policy review: South Africa’s progress in systematising its international and national responsibilities to protect, promote and support breastfeeding DST-NRF Centre of excellence in human development. DST-NRF Centre of Excellence in Human Development.

[24] Dinour LM, Szaro JM (2017). Employer-based programs to support breastfeeding among working mothers: a systematic review. Breastfeed Med.

[25] Chow T, Smithey Fulmer I, Olson BH (2011). Perspectives of managers toward workplace breastfeeding support in the state of Michigan. J Hum Lact.

[26] Burns E, Triandafilidis Z (2019). Taking the path of least resistance: a qualitative analysis of return to work or study while breastfeeding. Int Breastfeed J.

[27] Stratton JA, Henry BW (2011). What employers and health care providers can do to support breastfeeding in the workplace: aiming to match positive attitudes with action. ICAN: Infant, Child, & Adolescent Nutrition.

[28] Schaffer BS, Riordan CM (2003). A review of cross-cultural methodologies for organizational research: a best-practices approach. Organ Res Methods.

[29] Ollier-Malaterre A, Valcour M, Den Dulk L, Kossek EE (2013). Theorizing national context to develop comparative work–life research: a review and research agenda. Eur Manag J.

[30] Stumbitz B, Jaga A. A southern encounter: maternal body work and low-income mothers in South Africa. Gender, Work & Organization. 2020:1–16. 10.1111/gwao.12527 [Epub ahead of print].

[31] Lincoln YS, Guba EG (1986). But is it rigorous? Trustworthiness and authenticity in naturalistic evaluation. New Directions for Program Evaluation.

[32] Kendall M, Murray SA, Carduff E, Worth A, Harris F, Lloyd A (2009). Use of multiperspective qualitative interviews to understand patients’ and carers’ beliefs, experiences, and needs. Br Med J.

[33] Western Cape Government. *Socio-economic profile: City of Cape Town*. Cape Town:Western Cape Government. 2017. p. 1–41. Available from: https://www.westerncape.gov.za/assets/departments/treasury/Documents/Socio-economic-profiles/2017/city_of_cape_town_2017_socio-economic_profile_sep-lg_-_26_january_2018.pdf. Accessed 08 Oct 2020.

[34] Creswell JW, Creswell JD (2018). Research design: qualitative, quantitative, and mixed methods approaches. International student edition.

[35] Jackson K, Bazeley P (2019). Qualitative data analysis with NVivo: SAGE publications limited.

[36] Nowell LS, Norris JM, White DE, Moules NJ (2017). Thematic analysis: striving to meet the trustworthiness criteria. Int J Qual Methods.

[37] Braun V, Clarke V (2006). Using thematic analysis in psychology. Qual Res Psychol.

[38] Adhikari M (2009). Burdened by race: Coloured identities in Southern Africa: Uct press.

[39] Froh EB, Spatz DL (2016). Navigating return to work and breastfeeding in a hospital with a comprehensive employee lactation program: the voices of mothers. J Hum Lact.

[40] Trethewey A (1999). Disciplined bodies: women's embodied identities at work. Organ Stud.

[41] Lee R (2018). Breastfeeding bodies: intimacies at work. Gender, Work & Organization..

[42] Ashcraft KL (1999). Managing maternity leave: a qualitative analysis of temporary executive succession. Adm Sci Q.

[43] Carter P. Breast feeding and the social construction of heterosexuality, or ‘What breasts are really for’. Sex, sensibility and the gendered body. Palgrave Macmillan: Springer; 1996:99–119.

[44] Turner PK, Norwood K (2014). ‘I had the luxury . . .’: Organizational breastfeeding support as privatized privilege. Hum Relat.

[45] Kosmala-Anderson J, Wallace L (2006). Breastfeeding works: the role of employers in supporting women who wish to breastfeed and work in four organizations in England. J Public Health.

[46] Allen TD (2001). Family-supportive work environments: the role of organizational perceptions. J Vocat Behav.

[47] Cinamon RG (2006). Anticipated work-family conflict: effects of gender, self-efficacy, and family background. Career Dev Q.

[48] Greenhaus J, Beutell N (1985). Sources of conflict between work and family roles. Acad Manag Rev.

[49] West JM, Power J, Hayward K, Joy P (2017). An exploratory thematic analysis of the breastfeeding experience of students at a Canadian university. J Hum Lact.

[50] Johnston ML, Esposito N (2007). Barriers and facilitators for breastfeeding among working women in the United States.

[51] Dinour LM, Pope GA, Bai YK (2015). Breast milk pumping beliefs, supports, and barriers on a university campus. J Hum Lact.

